# Supplementary material for: Transport evidence of asymmetric spin–orbit coupling in few-layer superconducting 1Td-MoTe2
Source: Nat Commun. 2019 May 3;10:2044. doi: 10.1038/s41467-019-09995-0 (PMC6499809; doi:10.1038/s41467-019-09995-0)
Supplement: Supplementary file 1 — Supplementary Information [file 41467_2019_9995_MOESM1_ESM.docx]

**Supplementary Information**

**Transport evidence of asymmetric spin-orbit coupling in few-layer superconducting 1*T*_d_- MoTe_2_**

Jian Cui^1†^, Peiling Li^1,2†^, Jiadong Zhou^3†^, Wen-Yu He^4†^, Xiangwei Huang^1,2^, Jian Yi^5^, Jie Fan^1^, Zhongqing Ji^1^, Xiunian Jing^1,6^, Fanming Qu^1^, Zhi Gang Cheng^1^, Changli Yang^1,6^, Li Lu^1,6^, Kazu Suenaga^7^, Junwei Liu^4^, Kam Tuen Law^4^, Junhao Lin^8,9*^, Zheng Liu^3*^ and Guangtong Liu^1,10*^

^1^Beijing National Laboratory of Condensed Matter Physics, Institute of Physics, Chinese Academy of Sciences, Beijing 100190, China

^2^University of Chinese Academy of Sciences, Beijing 100049, China

^3^School of Materials Science and Engineering, Nanyang Technological University, Singapore 639798, Singapore

^4^Department of Physics, Hong Kong University of Science and Technology, Clear Water Bay, Hong Kong, China

^5^Ningbo Institute of Industrial Technology, Chinese Academy of Sciences, Ningbo 315201, China

^6^Collaborative Innovation Center of Quantum Matter, Beijing 100871, China

^7^National Institute of Advanced Industrial Science and Technology (AIST), Tsukuba 305-8565, Japan

^8^Department of Physics, Southern University of Science and Technology, Shenzhen 518055, China

^9^Shenzhen Key Laboratory of Quantum Science and Engineering, Shenzhen 518055, China

^10^Songshan Lake Materials Laboratory, Dongguan, Guangdong 523808, China

† These authors contributed equally to this work. Correspondence and requests for materials should be addressed to J.L (email: lin.junhao.stem@gmail.com), Z.L. (email: z.liu@ntu.edu.sg) and G.L. (email: gtliu@iphy.ac.cn)

**Supplementary Figures**

**
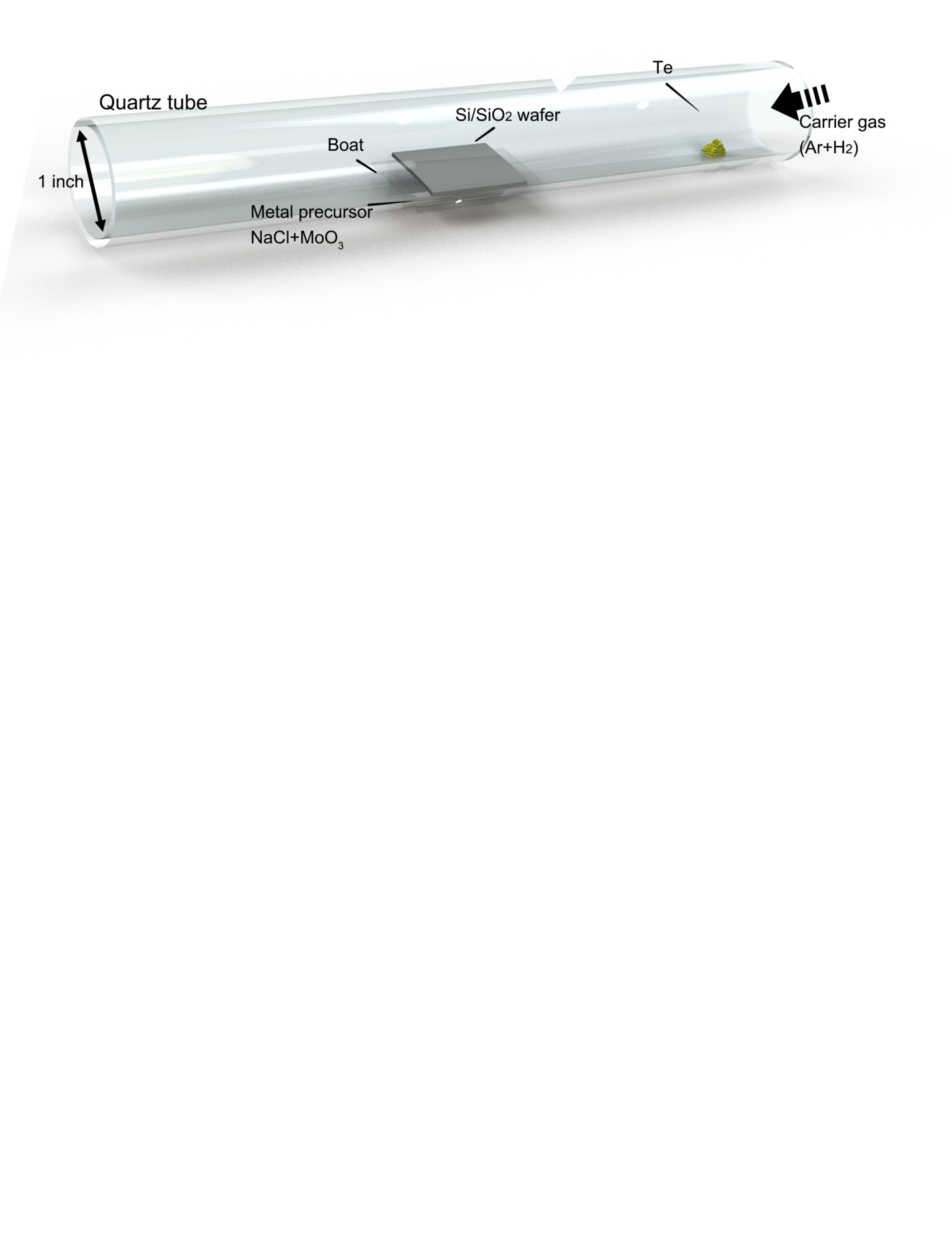
**

**Supplementary Figure 1.** The growth setup used for the controlled synthesis of few-layer MoTe_2_. In our experiment, the growth parameters were optimized to get better MoTe_2_ films. The mixed compounds (MoO_3_:NaCl =5:1) are placed in the center of the tube. Si/SiO_2_ substrate was placed on the alumina boat with the polished side faced down. Another alumina boat containing Te powder was put on the upstream side of quartz tube at a temperature of ~ 450 ^o^C. Mixed gas of H_2_/Ar with a flow rate of 15/80 sccm was used as the carrier gas. The furnace was ramped to 700 ^o^C at a rate of 50 ^o^C/min and held there for about 4 min to allow the growth of few-layer MoTe_2_ crystals. After the reaction, the temperature was naturally cooled down to room temperature. All reagents were purchased from Alfa Aesar with purity exceeding 99%. The thickness can be controlled by the growth time from 2 to 15 min.

**
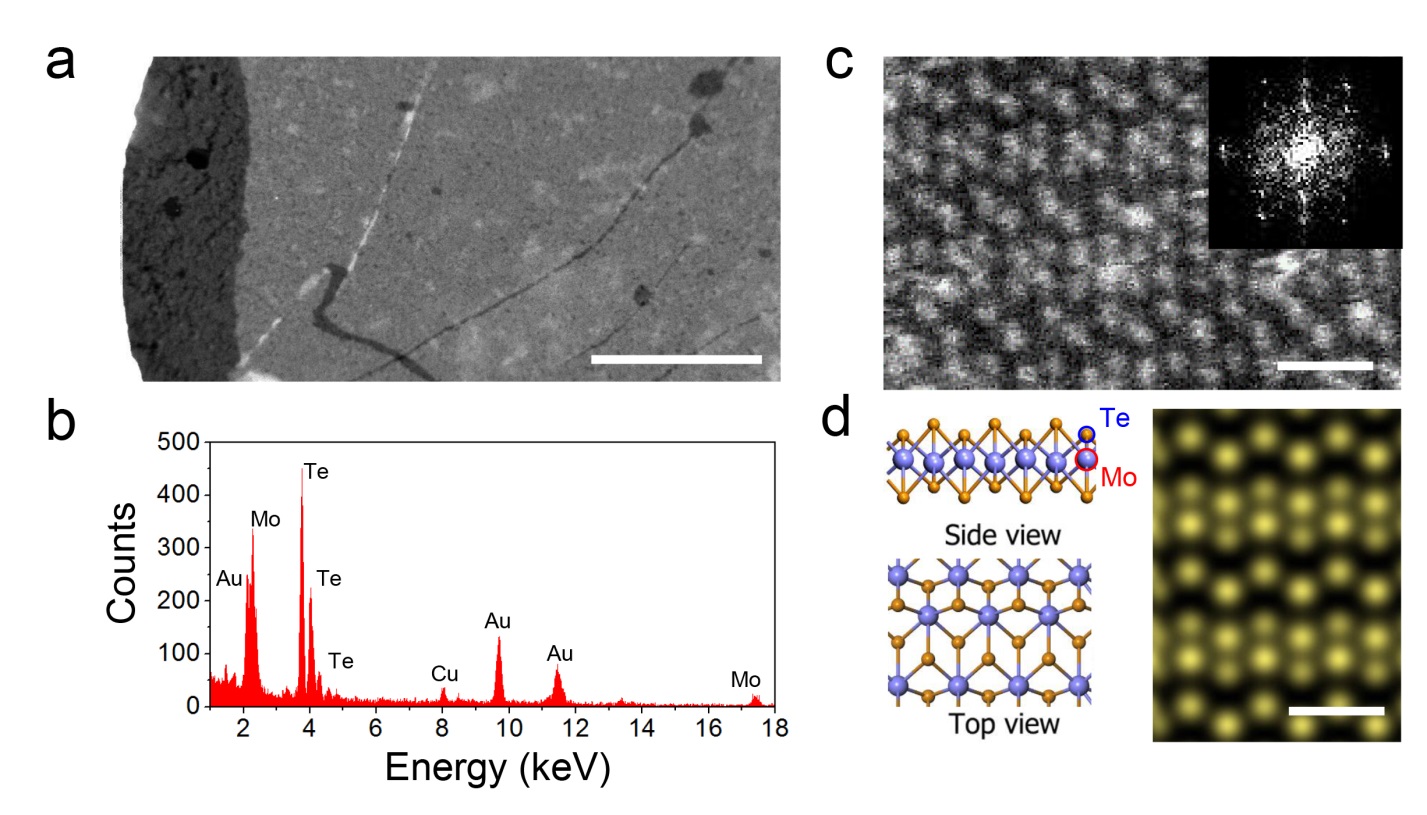
**

**Supplementary Figure 2. ADF-STEM image and EDX characterizations of the as-synthesized MoTe_2_ crystals. a,** Low magnified STEM image of a few-layer MoTe_2_ sample. The scale bar is 0.5 μm. **b,** Energy-dispersive X-ray spectrometry (EDX) spectrum of the few-layer MoTe_2_. The Au and Cu signal comes from the reflection of TEM grid bar and the pole piece. **c**, Atomic resolution ADF-STEM image of the monolayer MoTe_2_ region. Inset: FFT pattern of the image. The simulated STEM image and the top and side view of the atomic model are shown below, showing excellent agreement with the experiment. The scale bar is 0.5 nm.

**
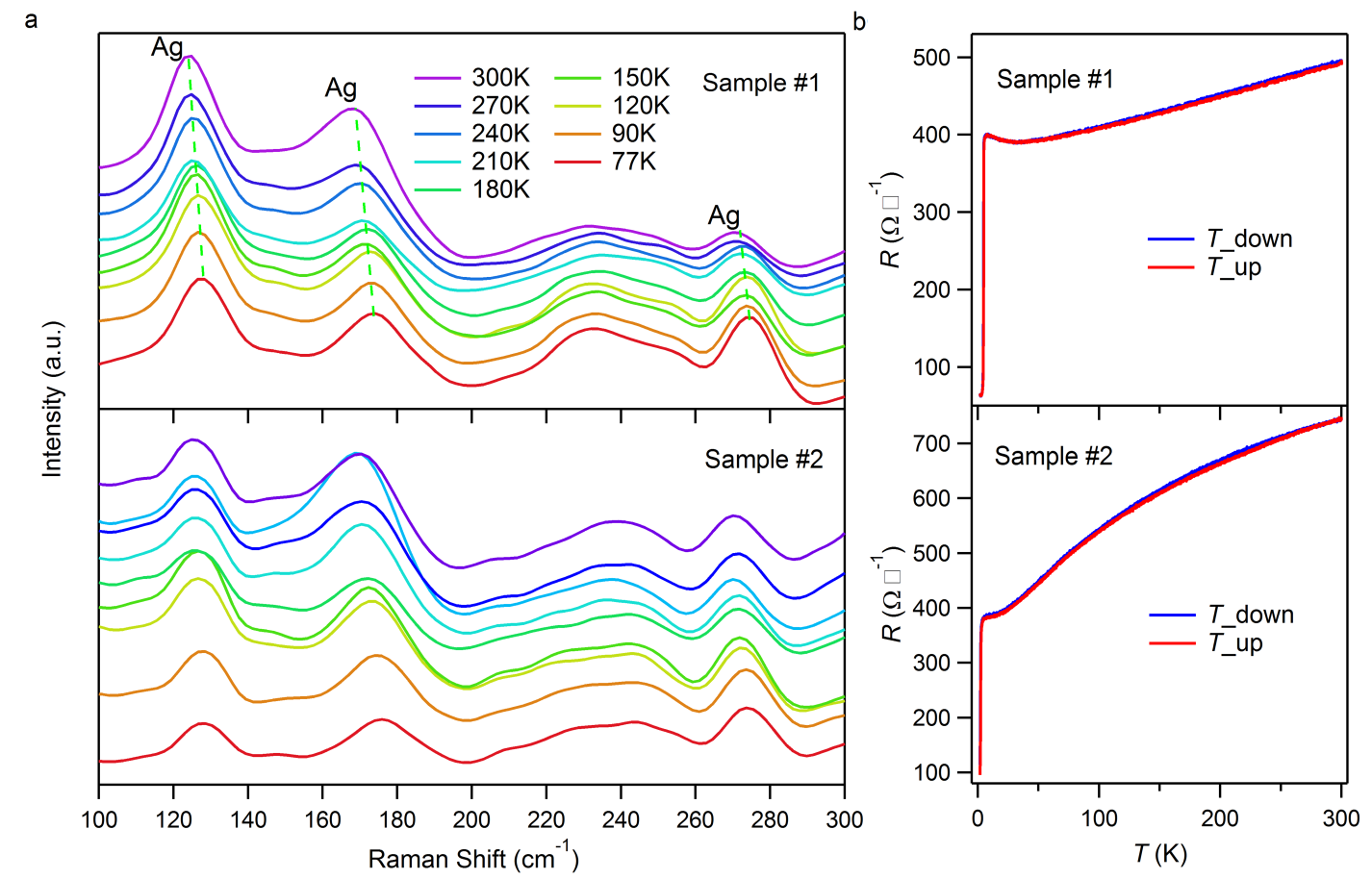
**

**Supplementary Figure 3. Characterization of few-layer 1*T*_d_-MoTe_2_. a,** Low-temperature Raman spectra for 7-nm-thick (Sample #1) and 9-nm-thick MoTe_2_ (Sample #2). Compared to the room-temperature Raman spectra shown in Fig. 1 in the main text, no extra modes can be discerned except that the Ag modes at 127, 161, and 267 cm^-1^ shift to low-frequency direction in low-temperature Raman spectra. Similar phenomenon was observed in mechanically exfoliated few-layered 1*T*_d_-MoTe_2_ samples^1^. **b,** Temperature dependence of sheet resistance for Sample #1 and Sample #2. Apparently for these two samples, no resistivity hysteresis was observed in *R*-*T* curves upon cooling and warming from 300 K to 2 K. This is distinct from its bulk counterpart, where a kink and a hysteresis always occur in *R*-*T* curves between cooling and warming around ∼250 K, which is interpreted as a signature of the 1*T*’-1*T*_d_ phase transition. Therefore, no phase transition occurs when the sample is cooled down. Combined with the observation of STEM, we can conclude that the 1*T*_d_ phase could be the intrinsic feature of the CVD-grown few-layer 1*T*_d_-MoTe_2_.


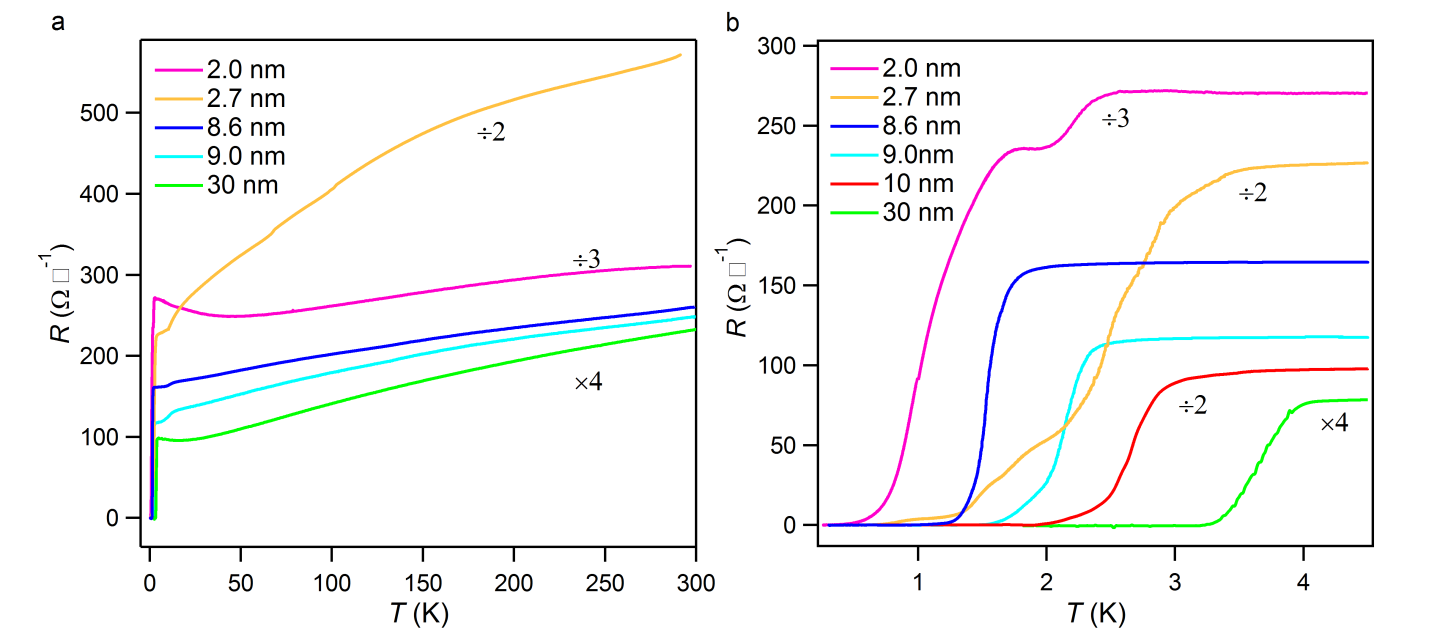


**Supplementary Figure 4.** Thickness dependence of the superconductivity in few-layer 1*T*_d_-MoTe_2_. **a,** Temperature dependence of sheet resistance of 1*T*_d_-MoTe_2_ with various thicknesses. For clarity, the data for 2-nm-thick and 2.7-nm-thick 1*T*_d_-MoTe_2_ were divided by 3 and 2, while the data was multiplied by 4 for 30-nm-thick MoTe_2_, respectively. **b**, Temperature dependence of sheet resistance of MoTe_2_ with various thicknesses in the range from 0.3 K to 4.5 K. For clarity, the data for 2-nm-thick, 2.7-nm-thick, and 10-nm-thick 1*T*_d_-MoTe_2_ were divided by 3, 2 and 2, while the data was multiplied by 4 for 30-nm-thick 1*T*_d_-MoTe_2_, respectively. Totally, a dozen of few-layer 1*T*_d_-MoTe_2_ samples were measured and all of them show metallic behavior at high temperatures before a superconducting transition emerging.

**
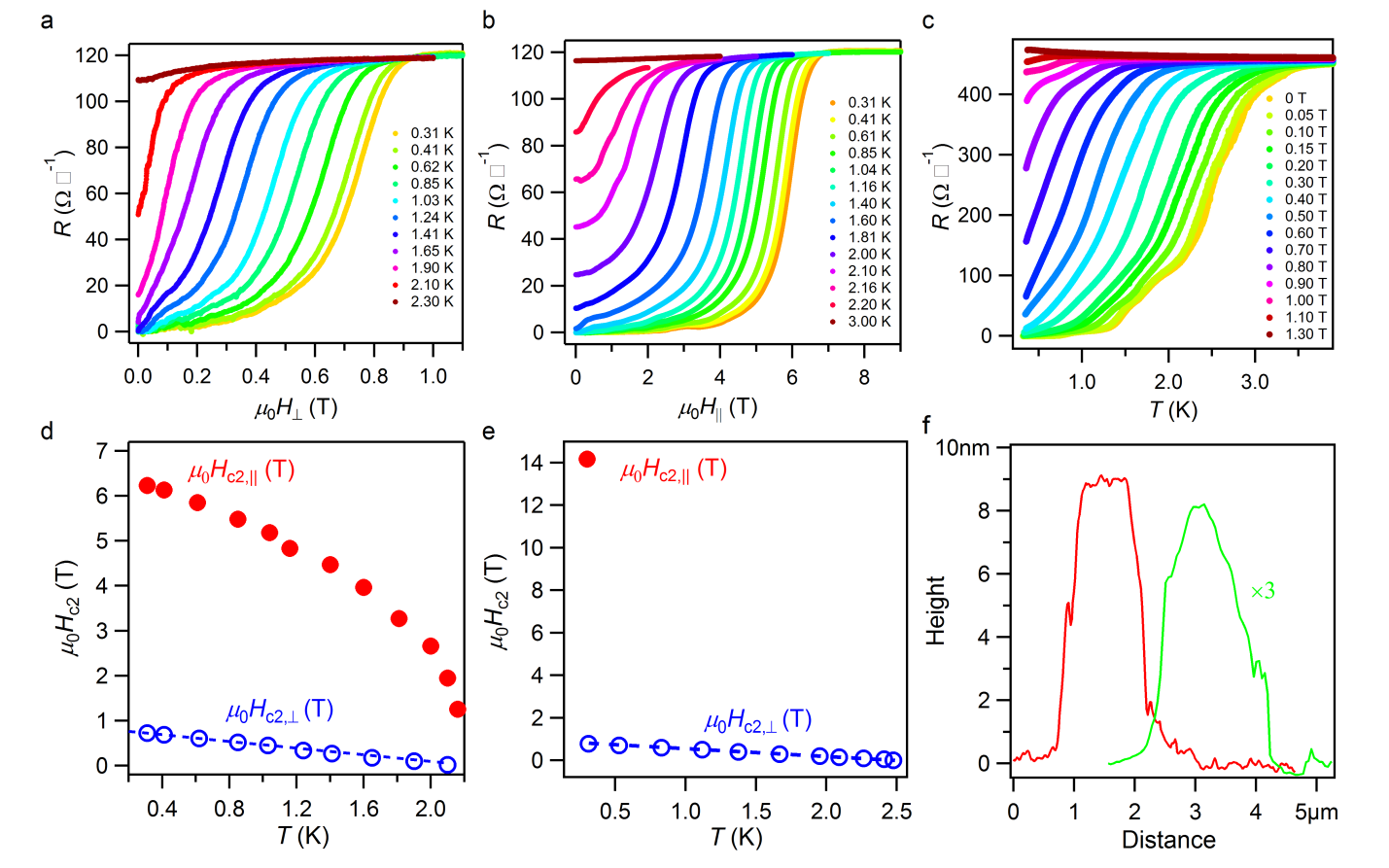
**

**Supplementary Figure 5. Two-dimensional superconductivity of few-layer 1*T*_d_-MoTe_2_ crystals.** Temperature dependence of superconducting resistive transition of the 9-nm-thick 1*T*_d_-MoTe_2_ crystal in perpendicular magnetic field (**a**) and in parallel magnetic field (**b**). **c,** Temperature dependence of superconducting resistive transition of the 2.7-nm-thick 1*T*_d_-MoTe_2_ crystal in perpendicular magnetic field. **d,** **e,** Temperature dependence of the upper critical field $\mu_{0}H_{c2,\parallel}(T)$ and $\mu_{0}H_{c2,\perp}\left( T \right)$, with magnetic field parallel and perpendicular to the crystal plane respectively, of the 9-nm-thick (**d**) and the 2.7-nm-thick (**e**) 1*T*_d_-MoTe_2_ crystals. Dashed lines are fittings to the 2D Ginzburg-Landau theory. **f,** AFM height profiles of two typical few-layer 1*T*_d_-MoTe_2_ crystals.

**
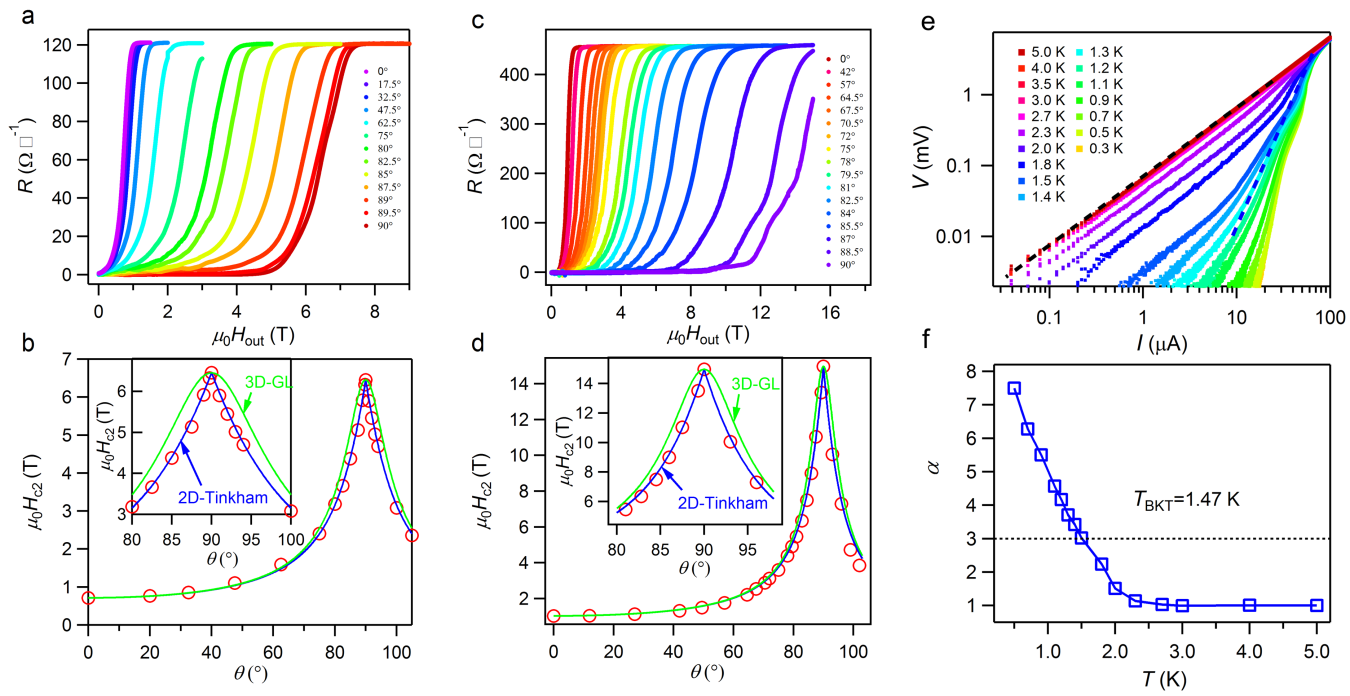
**

**Supplementary Figure 6. Two-dimensional superconductivity of few-layer 1*T*_d_-MoTe_2_**. **a, c,** Magnetic field dependence of the sheet resistance of a 9-nm-thick (**a**) and 2.7-nm-thick (**c**) 1*T*_d_-MoTe_2_ device at different tilted angles *θ* (the angle between a magnetic field and the normal of sample plane) at *T*=0.3 K. **b**, **d**, Angular dependence of the upper critical field *μ*_0_*H*_c2_ of a 9-nm-thick (**b**) and 2.7-nm-thick (**d**) 1*T*_d_-MoTe_2_ device. The solid lines represent the fitting with 2D Tinkham formula $\left| \frac{H_{c2}(\theta)\cos\theta}{H_{c2,\perp}} \right|+\left( \frac{H_{c2}(\theta)\sin\theta}{H_{c2,\parallel}} \right)^{2}=1$ (blue line) and 3D anisotropic mass model (3D-GL) $\left( \frac{H_{c2}(\theta)\cos\theta}{H_{c2,\perp}} \right)^{2}+\left( \frac{H_{c2}(\theta)\sin\theta}{H_{c2,\parallel}} \right)^{2}=1$ (green line), respectively. **e**, Voltage-current behavior (*V*-*I*) at different temperatures close to *T*_c_ plotted on a logarithmic scale. The black dashed line indicates the ohmic behavior expected in the normal state. The blue dashed line indicates $V\propto I^{3}$, which corresponds to *T*= *T*_BKT_. **f**, Temperature dependence of the exponent *α* obtained from fittings data in (**e**) to $V\propto I^{\alpha}$. *T*_BKT_=1.47 K is obtained from the intersection between the experimental curve and the dashed black line *α*=3.


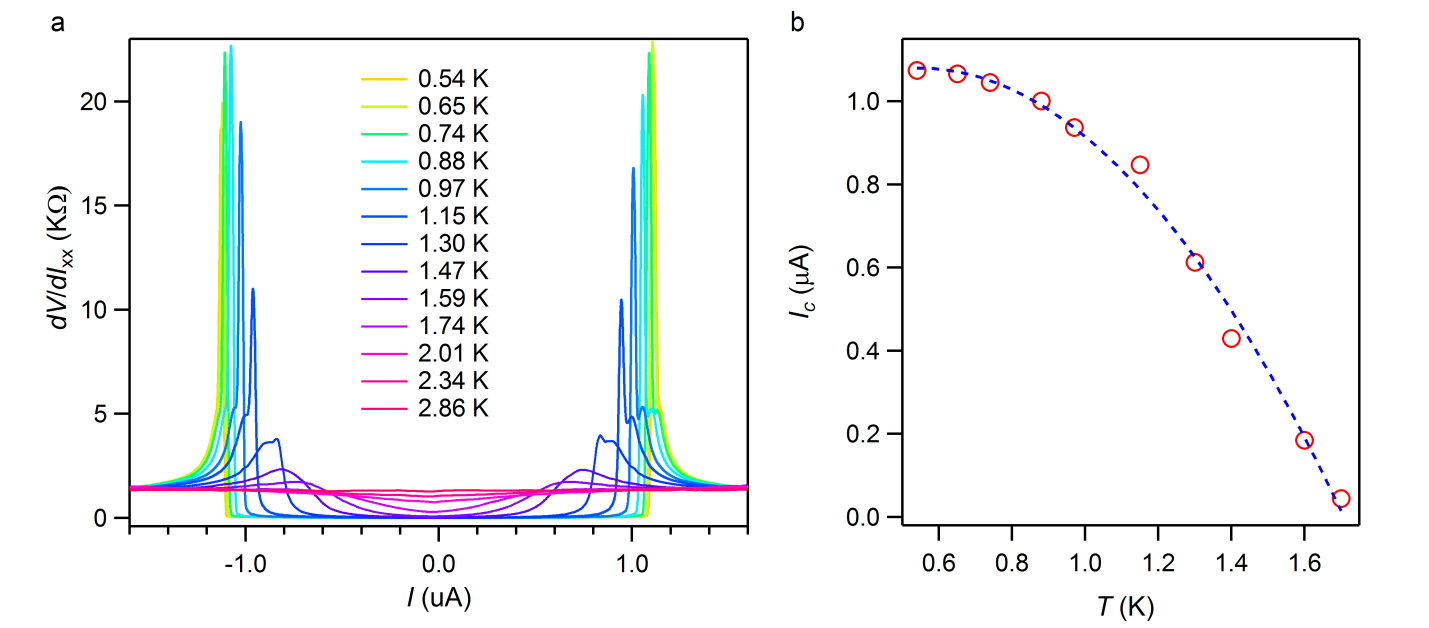


**Supplementary Figure 7. Characterization of superconductivity in few-layer 1*T*_d_-MoTe_2_. a,** Differential resistance of a 6.5-nm-thick 1*T*_d_-MoTe_2_ as a function of bias current at different fixed temperatures**.** The measurements were performed using a standard lock-in technique. The width of well-developed U-shape *dV*/*dI* curves narrows gradually with increasing temperature, and finally vanishes at ~2.0 K. **b**, Temperature dependence of the critical current *I*_c_ extracted from *dV*/*dI* curves. The dashed blue line is a guide to the eyes.


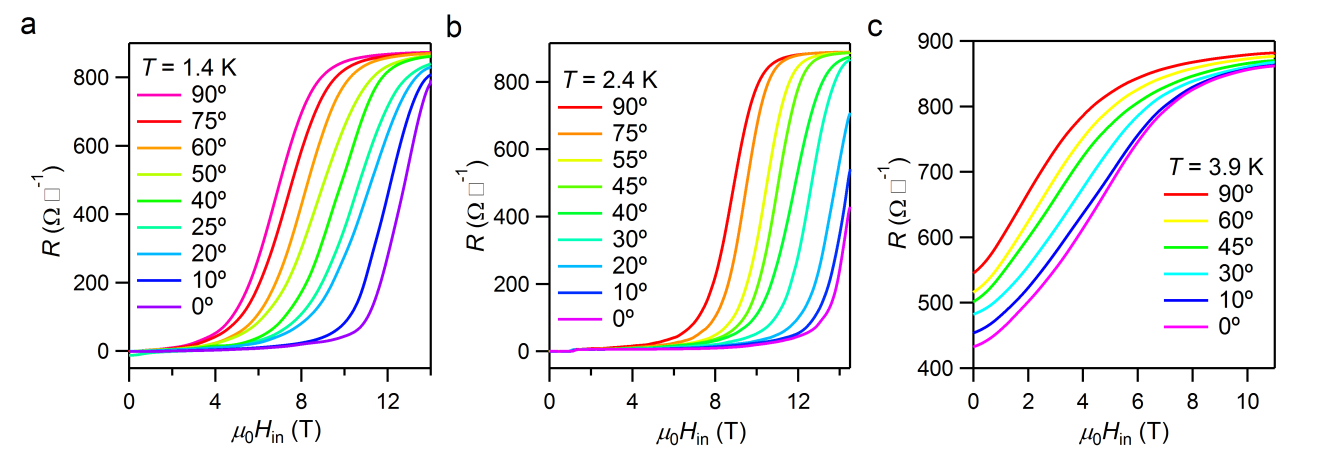


**Supplementary Figure 8.** Magnetic field dependence of the sheet resistance of the 3-nm-thick 1*T*_d_-MoTe_2_ device at *T* =1.4 K (*T* = 0.35 *T*_c_) (**a**), 2.4 K (*T* = 0.6 *T*_c_) (**b**), and 3.9 K (*T* = 0.95 *T*_c_) (**c**) with different in-plane tilted angles *φ* (*φ*, as shown in the inset of Fig. 2c, is the angle between the in-plane magnetic field and the positive direction of *y*-axis).


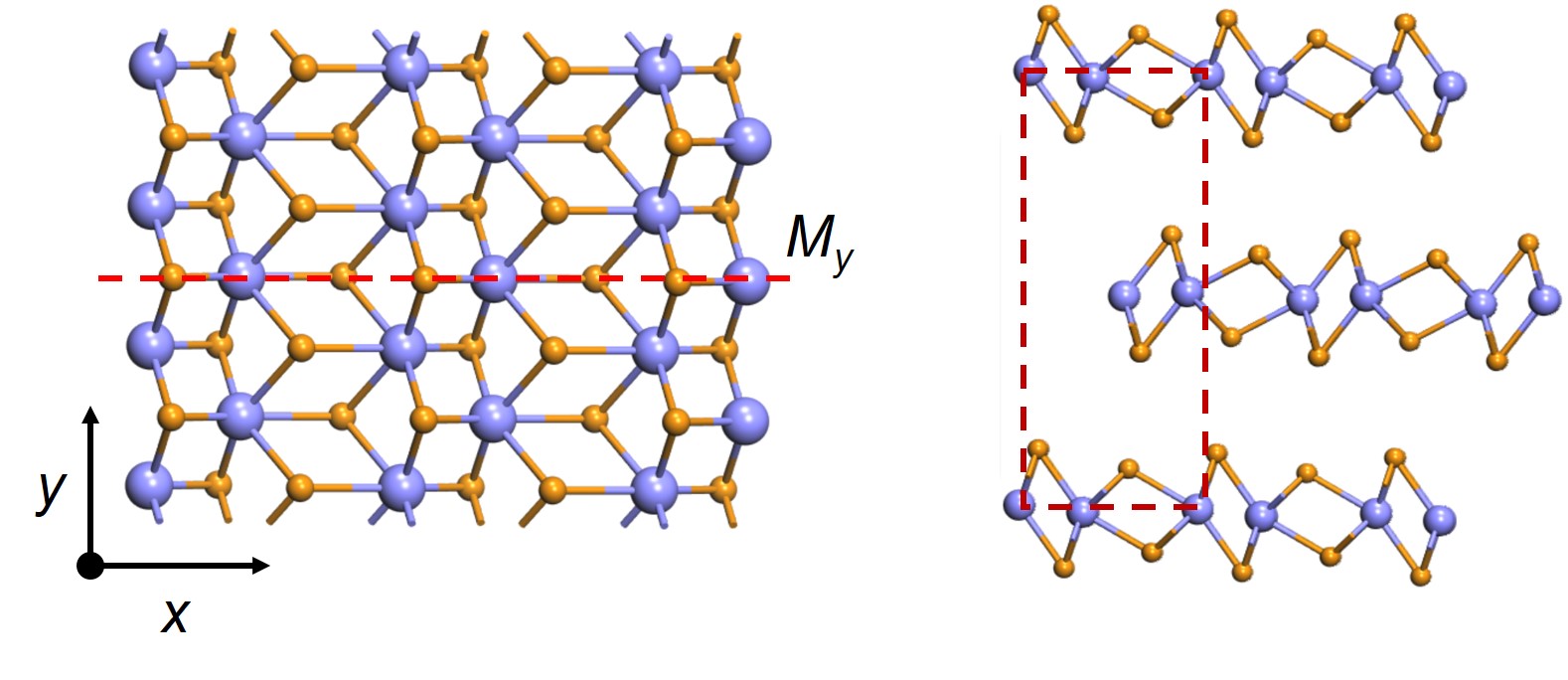


**Supplementary Figure 9. The crystal structure of the 1*T*_d_-MoTe_2_.** The left panel is the top view of the crystal structure and the right panel is the stacking order of the bilayer 1*T*_d_-MoTe_2_. The red dashed line denotes the mirror symmetry *M*_y_ for 1*T*_d_-MoTe_2_. In bilayer 1*T*_d_-MoTe_2_ the mirror symmetry *M*_y_ is respected but the stacking breaks its inversion symmetry.


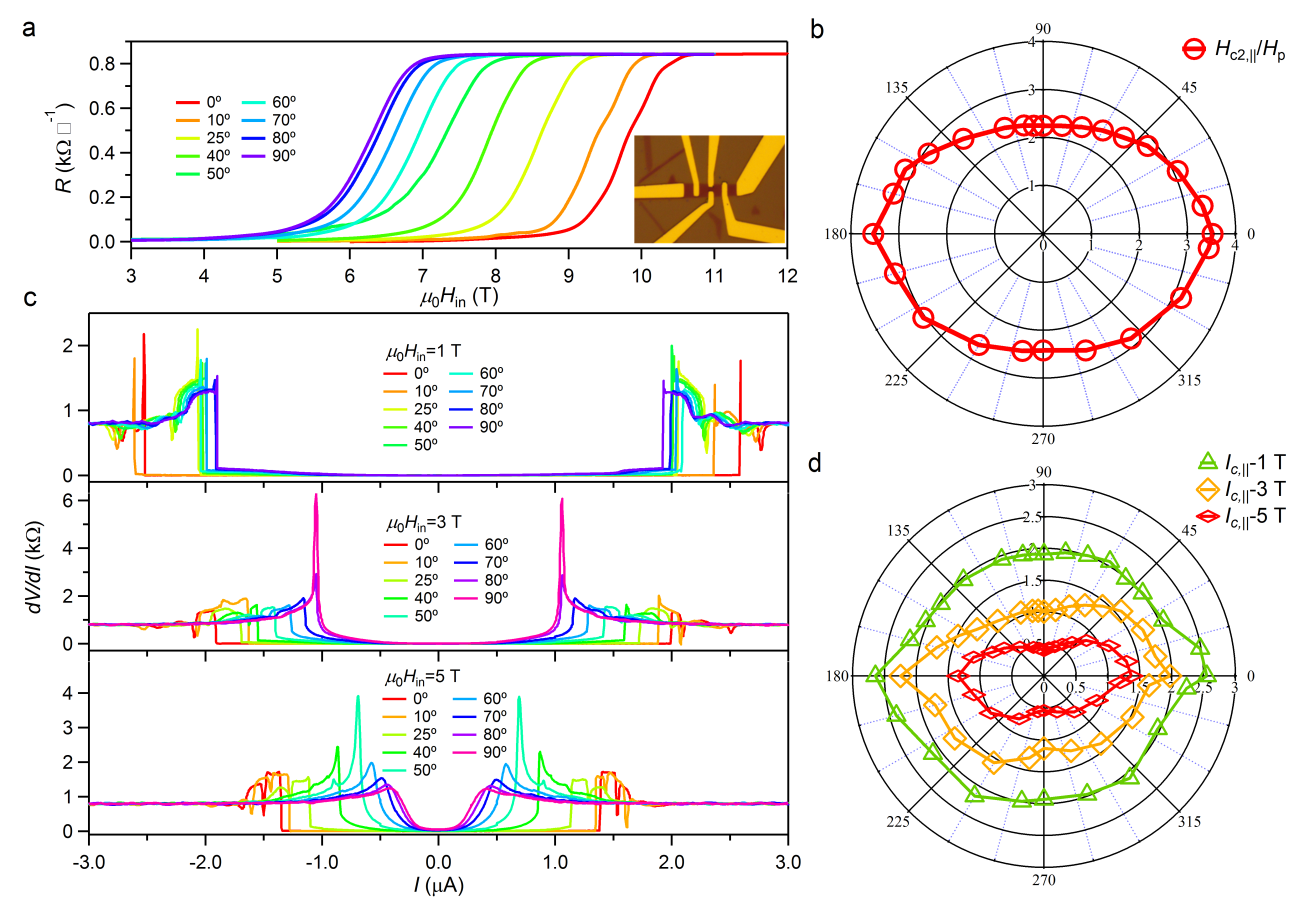


**Supplementary Figure 10. Two-fold symmetry in few-layer 1*T*_d_-MoTe_2_.** (**a**) Magnetic field dependence of the longitudinal resistance *R* of the 4-nm-thick MoTe_2_ device measured at *T* = 0.3 K (*T* = 0.2 *T*_c_) under typical in-plane tilted angles *φ*. Inset: Optical image of the 4-nm-thick MoTe_2_ device. (**b**) Angular dependence of the in-plane upper critical field normalized by Pauli limit ${H_{c2,\parallel}/H}_{P}$. (**c**) Tilted angle *φ* dependence of the in-plane critical current $I_{c,\parallel}(\varphi)$ measured at *T* = 0.3 K under in-plane field of 1 T, 3 T, and 5 T. (**d**) Angular dependence of the in-plane critical current $I_{c,\parallel}(\varphi)$ under in-plane field of 1 T, 3 T, and 5 T.


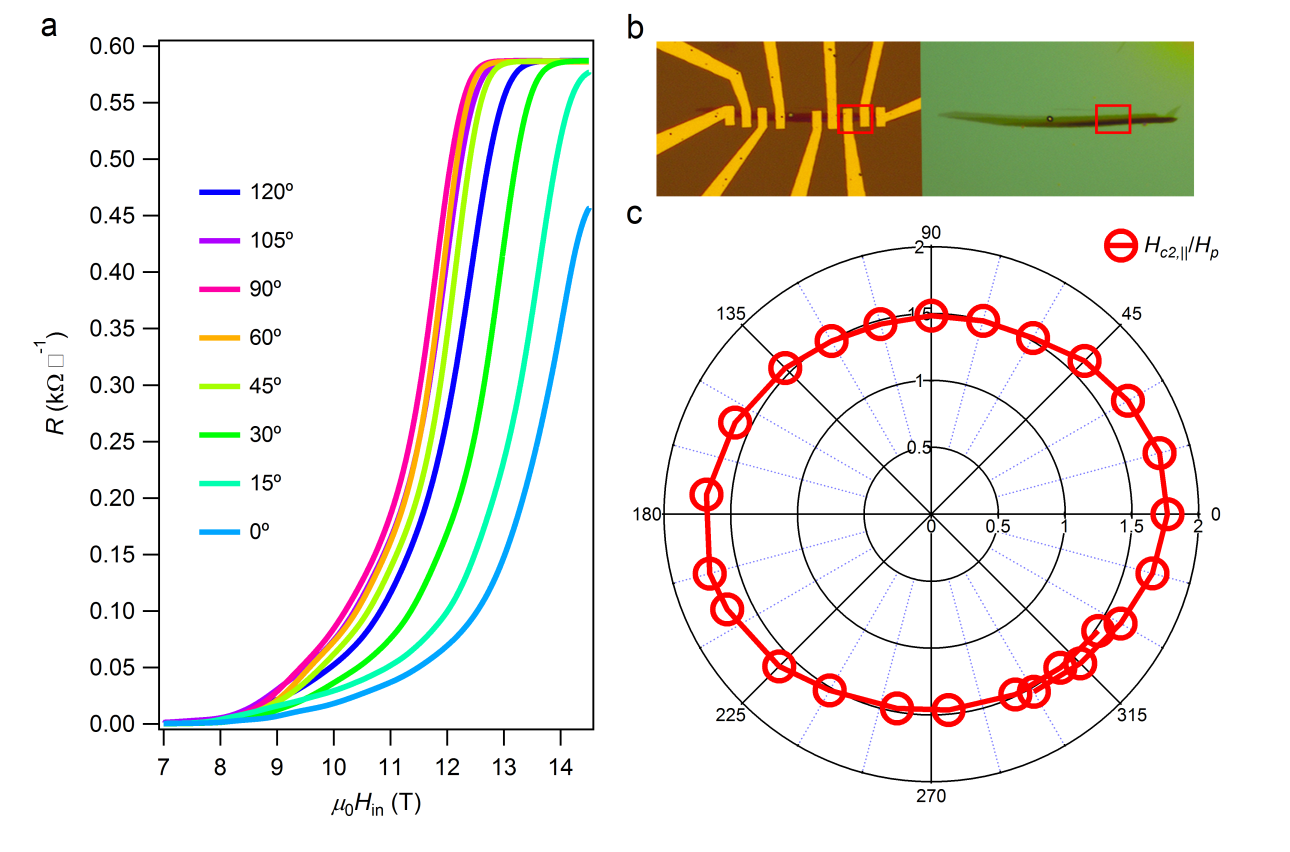


**Supplementary Figure 11.** **Two-fold symmetry in a thick 1*T*_d_-MoTe_2_ sample.** (**a**) Magnetic field dependence of the sheet resistance *R* of the 9-nm-thick MoTe_2_ device at *T* = 0.3 K (*T* = 0.07 *T*_c_) with typical in-plane tilted angles *φ*. (**b**) Optical image of the 9-nm-thick MoTe_2_ device. The red rectangular indicates the measured sample. (**c**) Angular dependence of the in-plane upper critical field normalized by Pauli limit ${H_{c2,\parallel}/H}_{P}$. Compared to Supplementary Figure 10b, though the two-fold symmetry can be observed, it is found that the in-plane anisotropy of the upper critical field represented by $H_{c2,\parallel}(0^{\circ})/H_{c2,\parallel}(90^{\circ})$ decreases sharply from 1.56 to 1.16 with the sample thickness increased from 4 nm to 9 nm, indicating that in-plane anisotropy strongly depends on the sample thickness. For the thicker samples, as superconductivity is also influenced by orbital effects, it’s reasonable that the anisotropy in *H*_c2,||_ is reduced.


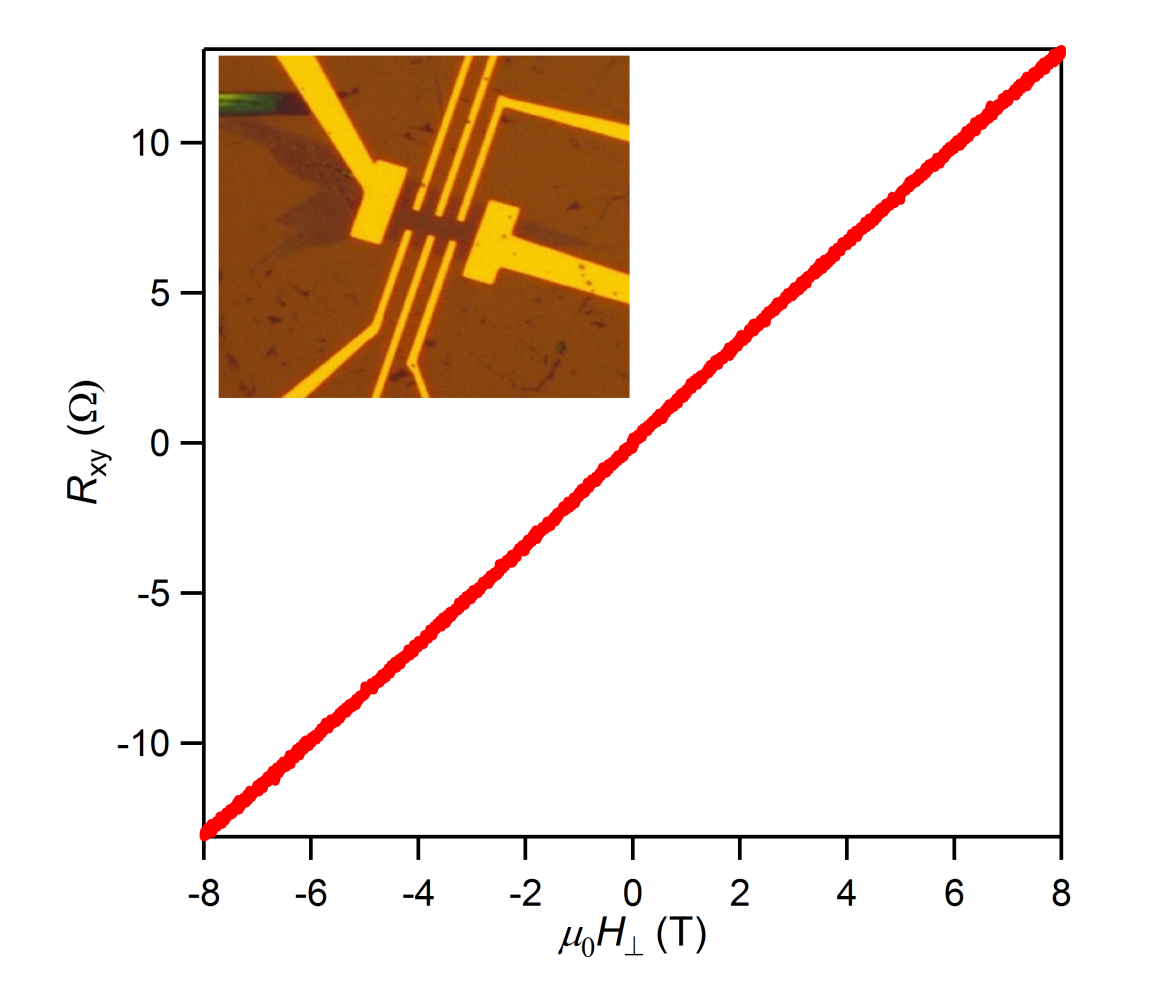


**Supplementary Figure 12.** Hall resistance *R*_xy_ measured at 10 K for a 3.0-nm-thick MoTe_2_ crystal. The inset shows the optical image of a typical Hall bar device. The data were collected in the normal state with the magnetic field perpendicular to the sample surface and with a current bias of 1 *μ*A. The carrier density and mean free path were estimated to be 2.52×10^14^ cm^-2^ and 10.3 nm by using the procedure as described in Supplementary Note 4.

**Supplementary Table 1.** Parameters of the measured 1*T*_d_-MoTe_2_ samples: Sample thickness (*d*), residual resistance ratio (RRR*)*, sheet resistance at *T*=5 K (*R*_5K_), and the set of parameters corresponding to individual reduced sheet resistance ($r$): critical temperatures ($T_{c,r}$), perpendicular (${\mu_{0}H}_{c2,\perp}$) and in-plane $(\mu_{0}H_{c2,\parallel})$upper critical fields, zero-temperature superconducting coherence length (*ξ*_GL_) and the superconducting thickness (*d*_sc_).

| *d* | RRR | *R*_5K_  (Ω□^-1^) | $r$ | $T_{c,r}$  (K) | *ξ*_GL_  (nm) | $\mu_{0}H_{c2,\parallel}$  (T) | *d*_sc_  (nm) |
| --- | --- | --- | --- | --- | --- | --- | --- |
| 2.0 nm | 1.15 | 810.0 | 0 | 0.35 | - | - | - |
|  |  |  | 0.5 | 1.12 | - | - | - |
|  |  |  | 0.9 | 2.14 | - | - | - |
| 2.7 nm | 2.51 | 454.6 | 0.05 | 1.31 | 25.40 | 10.67 | 2.69 |
|  |  |  | 0.5 | 2.56 | 19.33 | 14.30 | 3.86 |
|  |  |  | 0.9 | 3.12 | 17.55 | 15.30 | 4.04 |
| 3.0 nm | 2.17 | 637.0 | 0.05 | 1.61 | 28.38 | 13.30 | 2.72 |
|  |  |  | 0.5 | 2.32 | 19.29 | 19.69 | 2.80 |
|  |  |  | 0.9 | 2.92 | 17.17 | 23.38 | 2.69 |
| 6.5 nm | 0.76 | 488.2 | 0.05 | 1.15 | 18.79 | 5.80 | 8.99 |
|  |  |  | 0.5 | 1.92 | 16.42 | 8.50 | 7.50 |
|  |  |  | 0.9 | 2.57 | 13.97 | 10.10 | 7.60 |
| 8.6 nm | 1.61 | 163.7 | 0.05 | 1.10 | 30.95 | 4.14 | 7.81 |
|  |  |  | 0.5 | 1.54 | 20.79 | 5.38 | 9.15 |
|  |  |  | 0.9 | 1.71 | 19.73 | 6.68 | 8.05 |
| 9.0 nm | 2.10 | 118.5 | 0.05 | 1.50 | 30.45 | 3.88 | 8.75 |
|  |  |  | 0.5 | 2.13 | 19.70 | 5.87 | 9.13 |
|  |  |  | 0.9 | 2.32 | 18.83 | 6.40 | 8.83 |
| 10 nm | - | 196.0 | 0 | 1.90 |  | - | - |
|  |  |  | 0.5 | 2.66 |  | - | - |
|  |  |  | 0.9 | 2.92 |  | - | - |
| 30 nm | 2.36 | 19.70 | 0 | 3.16 |  | - | - |
|  |  |  | 0.5 | 3.65 |  | - | - |
|  |  |  | 0.9 | 3.91 |  | - | - |

*****RRR is defined by the ratio of the resistance at 300 K to the normal state resistance right above the superconducting transition, and $r=R/{R_{N}}=R/{R_{5K}}$. Values of ${\mu_{0}H}_{c2,\perp}$ and $\mu_{0}H_{c2,\parallel}$ corresponding to individual $r$ were extracted from magnetoresistance traces measured at *T*=0.3 K, and the *ξ*_GL_ and *d*_sc_ were estimated from $H_{c2,\perp}(T)=\frac{\phi_{0}}{2\pi\xi_{\mathrm{GL}}^{2}}\left( 1-\frac{T}{T_{c,0}} \right)$ and$H_{c2,\parallel}\left( T \right)=\frac{\phi_{0}\sqrt{12}}{2\pi\xi_{\mathrm{GL}}d_{\mathrm{sc}}}\sqrt{1-\frac{T}{T_{c,0}}}$ with *T*=0.3 K, respectively. Beyond 15 T the highest accessible magnetic field of our ^3^He system, the $H_{c2,\parallel}$ values shown in red color were obtained from extrapolation of the experimental data.

**Supplementary Notes**

**Supplementary Note 1**

**The symmetry analysis for the anisotropic spin-orbit coupling of the bilayer 1*T*_d_-MoTe_2_**

Since the bilayer *T*_d_-MoTe_2_ has no global inversion center, the spin-orbit coupling field induces spin splitting in the band structure. In general, at the Fermi level the Hamiltonian for the bilayer *T*_d_-MoTe_2_ can be written in the spin basis $\left[ c_{k_{F}\uparrow}, c_{k_{F}\downarrow} \right]$ as

$H_{0}=\xi\sigma_{0}+\mathbf{g}\cdot\boldsymbol{\sigma}$**,**

where ***σ***=(*σ_x_*, *σ_y_*, *σ_z_*) is the Pauli matrix and *σ_0_* is the identity matrix. The $\mathbf{g}\left( \varphi\right)$ is the spin-orbit coupling field for the Fermi circles with $\varphi$ the Fermi wave vector polar angle. It has the periodicity $2\pi$ so that its components can be expressed as the sum of Fourier series. As the bilayer *T*_d_-MoTe_2_ crystal structure respects the mirror symmetry along the *y* direction, the mirror symmetry $M_{y}=i\sigma_{y}$ and the time reversal symmetry $T=i\sigma_{y}K$ will generate restrictions on the Hamiltonian $H_{0}$ as

$$M_{y}^{-1}H_{0}\left( \varphi\right)M_{y}=H_{0}\left( -\varphi\right)$$

$T^{-1}H_{0}\left( \varphi\right)T=H_{0}\left( \pi-\varphi\right)$.

As a result, under the mirror symmetry $M_{y}=i\sigma_{y}$ the spin-orbit coupling field $\mathbf{g}\left( \varphi\right)=\left( g_{x}, g_{y}, g_{z} \right)$ satisfies

$$g_{x}\left( \varphi\right)=-g_{x}\left( -\varphi\right)$$

$$g_{y}\left( \varphi\right)=g_{y}\left( -\varphi\right)$$

$g_{z}\left( \varphi\right)=-g_{z}\left( -\varphi\right)$,

and under the time reversal symmetry $T=i\sigma_{y}K$, the spin-orbit coupling field $\mathbf{g}\left( \varphi\right)=\left( g_{x}, g_{y}, g_{z} \right)$ satisfies

$$g_{x}\left( \varphi\right)=-g_{x}\left( \varphi+\pi\right)$$

$$g_{y}\left( \varphi\right)={-g}_{y}\left( \varphi+\pi\right)$$

$g_{z}\left( \varphi\right)=-g_{z}\left( \varphi+\pi\right)$.

Under both the mirror symmetry $M_{y}=i\sigma_{y}$ and the time reversal symmetry $T=i\sigma_{y}K$, the spin-orbit coupling field $\mathbf{g}\left( \varphi\right)$ is restricted to the form

$g_{x}\left( \varphi\right)=\sum_{0}^{\infty} x_{n}\sin\left( 2n-1 \right)\varphi$,

$g_{y}\left( \varphi\right)=\sum_{0}^{\infty} y_{n}\cos\left( 2n-1 \right)\varphi$,

$g_{z}\left( \varphi\right)=\sum_{0}^{\infty} z_{n}\sin\left( 2n-1 \right)\varphi$.

Here we take the spin-orbit coupling field $\mathbf{g}\left( \varphi\right)$ up to the first order $n=1$ term for the further calculation to fit the experimental results.

**Supplementary Note 2**

**The in-plane spin susceptibility**

The spin susceptibility in the superconducting state can be calculated by means of Green’s function as^2^

$\chi_{ij}^{s}=-\mu_{B}^{2}k_{B}T\sum_{\boldsymbol{k}} \sum_{\omega_{n}} \mathrm{tr} \left[ \sigma_{i}G\left( \boldsymbol{k},\omega_{n} \right)\sigma_{j}G\left( \boldsymbol{k},\omega_{n} \right)-\sigma_{i}F\left( \boldsymbol{k},\omega_{n} \right)\sigma_{j}^{T}F\left( \boldsymbol{k},\omega_{n} \right) \right]$,

where $\sigma_{i}$ is the matrix representation for spins along the *i*-direction, $G\left( \boldsymbol{k},\omega_{n} \right)$ and $F\left( \boldsymbol{k},\omega_{n} \right)$ are the normal and anomalous Green’s function respectively defined as

$$\left( \begin{matrix} G\left( \boldsymbol{k},\omega_{n} \right) & F\left( \boldsymbol{k},\omega_{n} \right) \\ -F^{\dagger}\left( \boldsymbol{k},\omega_{n} \right) & {-G}^{T}\left( \boldsymbol{-k},-\omega_{n} \right) \end{matrix} \right)=\frac{1}{i\omega_{n}-\xi\tau_{z}\sigma_{0}-g_{x}\tau_{0}\sigma_{x}-g_{y}\tau_{z}\sigma_{y}-g_{z}\tau_{0}\sigma_{z}-\Delta\tau_{y}\sigma_{y}}.$$

with $\omega_{n}$ the Matsubara frequency. In the Fermi pocket we can replace the sum over ***k*** by $\sum_{\boldsymbol{k}} \to N_{0}\int\frac{d\varphi}{2\pi}d\xi$, where $N_{0}$ is the density of states at the Fermi level and then the in-plane spin susceptibility along the direction with polar angle $\varphi$ reads

$\chi_{\varphi\varphi}^{s}=\chi_{n}\left\{ 1-\pi k_{B}T\sum_{\omega_{n}} \int_{0}^{2\pi} \frac{\Delta^{4}+\Delta^{2}\omega_{n}^{2}+\frac{1}{2}\left[ \left( 1+\cos2\varphi\right)g_{x}^{2}+\left( 1-\cos2\varphi\right)g_{y}^{2}+2g_{x}g_{y}\sin2\varphi\right]}{\left( \Delta^{2}+\omega_{n}^{2} \right)^{\frac{3}{2}}\left( g_{x}^{2}+g_{y}^{2}+g_{z}^{2}+\Delta^{2}+\omega_{n}^{2} \right)}\frac{d\varphi}{2\pi} \right\}$.

Near *T*=0 K, the in-plane upper critical field *H*_c2_ can be approximated as

$$H_{c2,\parallel}(\varphi)=\sqrt{\frac{N_{0}}{\chi_{n}-\chi_{\varphi\varphi}^{s}}}\Delta$$

As a result, given the experimentally measured $H_{c2,\parallel}(\varphi)$, we can tune the spin-orbit coupling field $\mathbf{g}\left( \varphi\right)$ to get the optimized to in-plane spin susceptibility $\chi_{\varphi\varphi}^{s}$ that can fit the $H_{c2,\parallel}(\varphi)$ data at *T* = 0.07 *T*_c_. After the optimization process, we get the fitted spin-orbit coupling field $\mathbf{g}=k_{B}T_{c}\left( 49\sin\varphi,68\cos\varphi,67\sin\varphi\right)$.

**Supplementary Note 3**

**Superconducting gap as a function of in-plane magnetic field**

In the presence of an in-plane magnetic field, the Bogliubov-de Gennes Hamiltonian for the spin-orbit coupling system can be written as

$H=\xi\tau_{z}\sigma_{0}+g_{x}\tau_{0}\sigma_{x}+g_{y}\tau_{z}\sigma_{y}+g_{z}\tau_{0}\sigma_{z}+\Delta\tau_{y}\sigma_{y}+\frac{1}{2}g\mu_{B}\left( \tau_{z}H_{x}\sigma_{x}+\tau_{0}H_{y}\sigma_{y} \right)$.

The superconducting gap $\Delta$ as a function of the in-plane magnetic field can be determined through minimizing the free energy density

$F=\frac{\Delta^{2}}{U}-\frac{N_{0}}{V}\sum_{n} \int_{0}^{\omega_{D}} \int_{0}^{2\pi} \frac{k_{B}T}{2}\ln\left( 1+e^{-\frac{E_{n}}{k_{B}T}} \right)\frac{d\phi}{2\pi}d\xi$,

where $E_{n}$ is the eigen-energy of the Bogliubov-de Gennes Hamiltonian and $\omega_{D}$ is the Debye frequency.

**Supplementary Note 4**

**Estimation of carrier density *n*_s_**

By fitting the experimental data in Supplementary Figure 10, we can calculate the carrier density *n_s_* by:

$$n_{s}=\frac{I/e}{dV_{H}/dB}=\frac{1/e}{dR_{H}/dB}=2.52\times{10}^{14} \mathrm{cm}^{-2}$$

**Estimation of sheet resistance *R*_s_**

The width *w* and length *l* for the measured sample can be determined by the optical image, *l*=1.84 *μ*m, w=1.75 *μ*m; The normal state resistance *R*_N_=669 Ω was taken at *T*=10.0 K. The sheet resistance *R*_s_ can be calculated by:

$$R_{s}=\frac{R_{N}w}{l}=637 \Omega\square^{-1}$$

**Estimation of mobility *μ***

According to the Drude model, the mobility *μ* can be calculated by:

$$\mu=\frac{1}{eR_{s}n_{s}}=38.8\mathrm{cm}^{2}/\mathrm{Vs}$$

**Estimation of the Fermi velocity *v*_F_**

In two-dimensional systems, the Fermi velocity *v*_F_ can be calculated by:

$$\nu_{F}=\frac{\hbar k_{F}}{m}=\frac{\hbar}{m}\sqrt{2\pi n_{s}}=4.6\times{10}^{5} m/s$$

**Estimation of the momentum scattering time *τ*_m_**

$$\tau_{m}=\frac{\mu m}{e}=2.2\times{10}^{-14} s$$

**Estimation of the mean free path *l*_m_**

$$l_{m}=\nu_{F}\tau_{m}=\frac{\hbar k_{F}}{e}\mu=10.3 \mathrm{nm}$$

**Supplementary Reference**

1. He, R. *et al*. Dimensionality-driven orthorhombic MoTe_2_ at room temperature. *Phys. Rev.B* **97**, 041410 (R) (2018).
2. Frigeri, P. A. *et al*. Spin susceptibility in superconductors without inversion symmetry. *New J. Phys*. **6**, 115 (2004).
